# Integration of microRNAome, proteomics and metabolomics to analyze arsenic-induced malignant cell transformation

**DOI:** 10.18632/oncotarget.18741

**Published:** 2017-06-27

**Authors:** Youyou Zhou, Yanfu Wang, Juan Su, Zheng Wu, Chao Wang, Weiming Zhong, Xiaomei Liu, Linhui Cui, Xiaoyu Zhou, Yufang Ma, Yi Xin, Jianglin Zhang, Lisha Wu, Xing Hu, Xiang Chen, Cong Peng, MingYang Gao

**Affiliations:** ^1^ Department of Dermatology, Xiangya Hospital, Central South University, Changsha 410008, Hunan, China; ^2^ Department of Dermatology, The First Affiliated Hospital of Dalian Medical University, Dalian 116011, Liaoning, China; ^3^ Department of Neurosurgery, Xiangya Hospital, Central South University, Changsha 410008, Hunan, China; ^4^ Department of Gerontology, The First Affiliated Hospital of Dalian Medical University, Dalian 116011, Liaoning, China; ^5^ Department of Biochemistry and Molecular Biology, Dalian Medical University, Dalian 116044, Liaoning, China

**Keywords:** arsenic, microRNAome, proteomics, metabolomics, cutaneous squamous cell carcinoma

## Abstract

Long-term exposure to arsenic has been linked to tumorigenesis in different organs and tissues, such as skin; however, the detailed mechanism remains unclear. In this present study, we integrated “omics” including microRNAome, proteomics and metabolomics to investigate the potential molecular mechanisms. Compared with non-malignant human keratinocytes (HaCaT), twenty-six miRNAs were significantly altered in arsenic-induced transformed cells. Among these miRNAs, the differential expression of six miRNAs was confirmed using Q-RT-PCR, representing potential oxidative stress genes. Two-dimensional gel electrophoresis (2D-PAGE) and mass spectrometry (MS) were performed to identify the differential expression of proteins in arsenic-induced transformed cells, and twelve proteins were significantly changed. Several proteins were associated with oxidative stress and carcinogenesis including heat shock protein beta-1 (HSPB1), peroxiredoxin-2 (PRDX2). Using ultra-performance liquid chromatography and Q-TOF mass spectrometry (UPLC/Q-TOF MS), 68 metabolites including glutathione, fumaric acid, citric acid, phenylalanine, and tyrosine, related to redox metabolism, glutathione metabolism, citrate cycle, met cycle, phenylalanine and tyrosine metabolism were identified and quantified. Taken together, these results indicated that arsenic-induced transformed cells exhibit alterations in miRNA, protein and metabolite profiles providing novel insights into arsenic-induced cell malignant transformation and identifying early potential biomarkers for cutaneous squamous cell carcinoma induced by arsenic.

## INTRODUCTION

The worldwide metalloid arsenic pollution is a complex global environmental challenge. Humans are frequently exposed to arsenic as a natural environmental pollutant of water, air, soil and food [1]. Several epidemiological studies have associated arsenic exposure with increasing incidence of skin, liver, bladder, and lung cancer [2, 3]. This issue has been exacerbated by an increment in the use of arsenic-contaminated water, particularly in developing countries [4]. Therefore, inorganic arsenic has been classified as human carcinogen based on strong epidemiological data [5–7].

The effects of arsenic exposure on skin have been documented, particular in skin cancer [8, 9]. Arsenic-induced skin lesions are characteristic by veracious hyperkeratosis and pigmentation disorders [10]. Other skin lesions associated with human arsenic exposure involves Bowen’s disease and squamous cell or basal cell carcinoma [11–14]. Sarkar et al. showed that arsenic may exert toxicity via oxidative stress, indicated by related metabolic biomarkers [15]. However, these studies only focused on one or two aspects of arsenic-driven skin carcinogenesis and a global view of arsenic effects is lacking.

MicroRNAs (miRs) are endogenous 22 nt noncoding RNAs that bind to the untranslated region of target mRNAs and regulate messenger translation through mRNA cleavage, translational repression or mRNA destabilization [16–18]. Micro-RNA exerts critical physiological functions [19–31]. Proteins are the primary effector molecules of all living systems, and any adaptive responses to exotic stresses are reflected through alterations in protein activity or content [32]. Metabolic patterns, the endpoints of enzymatic (protein) activity, are the final consequence of biological function and directly indicate aberrant physiological status.

‘Omics’ technologies, including microRNAome, proteomics and metabolomics, which provide information on global profile, are therefore regarded as powerful tools to investigate toxic responses to environmental pollutant exposure compared with conventional endpoint bioassays [33–35]. In addition, the integration of microRNAome, proteomics and metabolomics profiles may provide increased reliability in interpreting metabolic alterations resulting from certain genes or proteins, enabling further elucidation of the toxicological effects [36]. Although previous reports have addressed the effects of arsenic exposure on skin using high throughput technology [37–43], integrated microRNAome, proteomics and metabolomics profiles to study skin carcinogenesis induced by arsenic have not been reported.

HaCaT cell lines have been malignant transformed by continuous exposure to environmentally relevant levels of inorganic arsenic (100 nM) for 28 weeks [44]. In the present study, we generated an arsenic-induced cell transformation model, and integrated microRNAome, proteomics, and metabolomics approaches to investigate alterations in the cellular profiles in arsenic-transformed cells (AS-TM), providing important insights into the initial molecular response to arsenic exposure.

## RESULTS

### Low dosage of arsenic induces HaCaT cells malignant transformation

**Figure 1:**
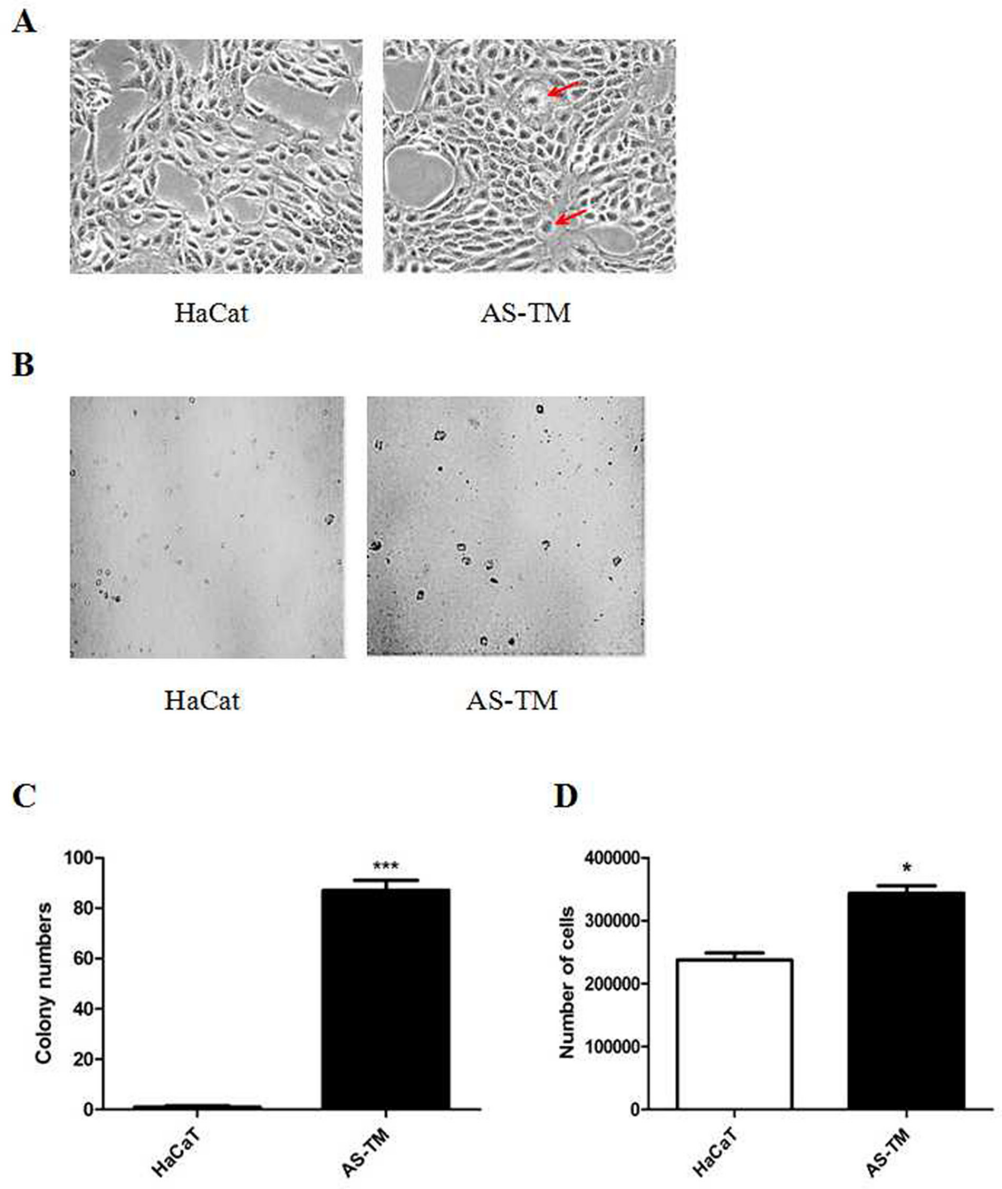
Low-dosage arsenic treatment induces malignant transformation HaCaT cells were continuously maintained in DMEM medium containing 100 nM of sodium arsenite (NaAsO_2_) for 28 weeks. **(A)** Morphological alterations in arsenic-exposed cells. The images were captured using a microscope with a camera (200× magnification), and the arrows indicate giant multinuclear cells. **(B** and **C)** Colony-formation growth in soft agar. Arsenic-treated and control cells were seeded onto 0.3% BME agar containing 10% FBS. The cultures were maintained in a 37°C, 5% CO_2_ incubator for 10 days, and subsequently the colonies were counted using a microscope (100× magnification) and the ImageJ computer software program. **(D)** The number of HaCaT cells compared with arsenic-treated cells at 24 h after passage (mean ± SEM, n=3), * p < 0.05 difference from passage control cells. The data from multiple experiments are expressed as the mean ± S.D. Significant differences were evaluated using one-way ANOVA, and the respective significant differences are as indicated, p < 0.001 difference from passage control cells. AS-TM: arsenite-transformed cells.

To achieve oncogenic transformation, HaCaT cells were continuously exposed to a low level (100 nM) of inorganic arsenite. After 28 weeks of continuous arsenic exposure, the arsenic-treated cells (AS-TM) exhibited unique morphological alterations with the frequent occurrence of giant multinuclear cells (Figure 1A), which are common during malignant transformation and in tumors [45]. The AS-TM cell proliferation was significantly promoted after malignant transformation, as shown in Figure 1D. Anchorage-independent growth is a characteristic of transformed cells [45]. To determine whether cells chronically exposed to arsenite acquired this capacity, anchorage-independent growth was examined and the results showed that HaCaT cells exposed to 100 nM sodium arsenic could undergo colony growth in agar compared with control cells (Figure 1B and 1C). Therefore, cells chronically exposed to this low level of arsenite acquired a malignant phenotype.

### Arsenic exposure induced the differential expression of miRNAs

**Figure 2:**
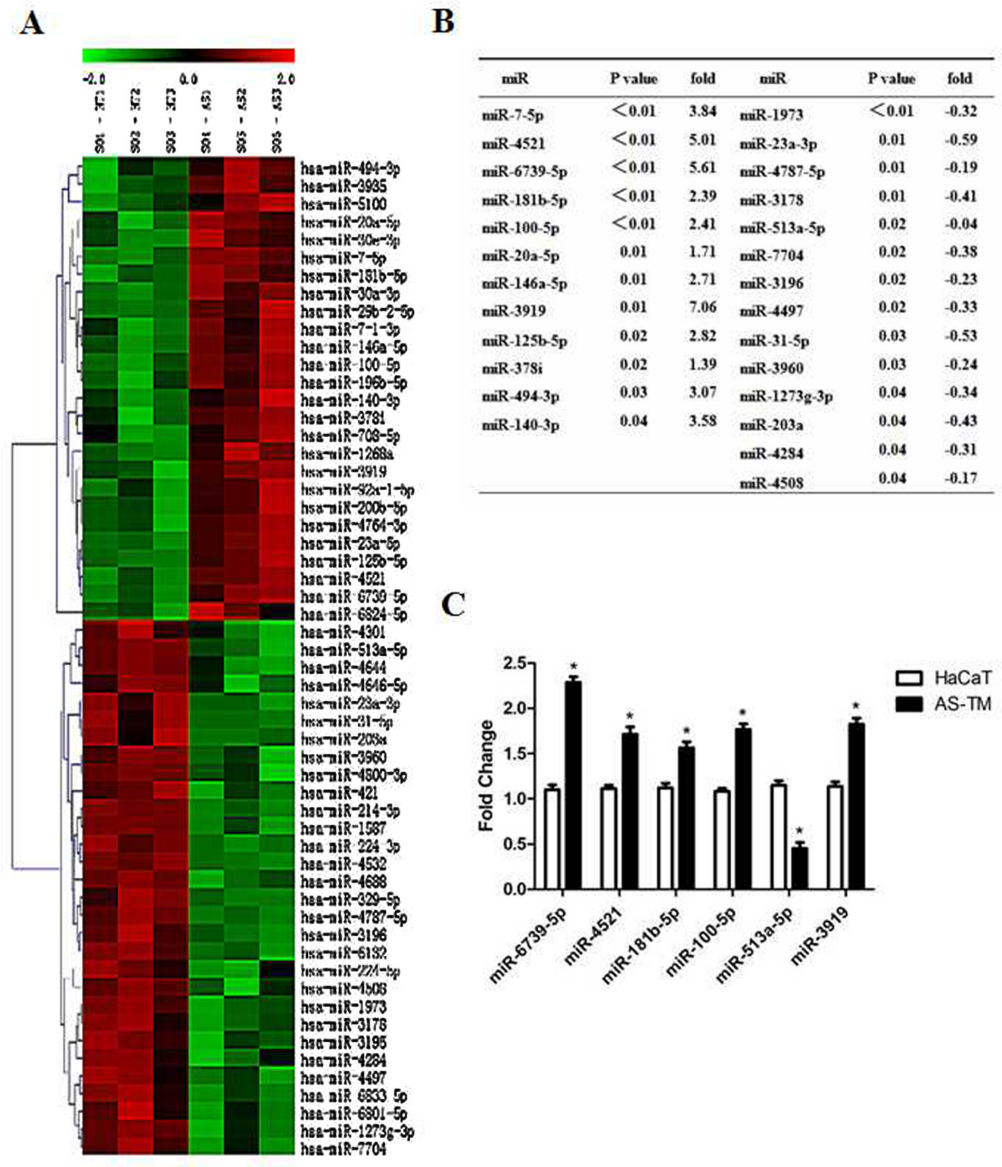
Arsenic exposure induced the differential expression of miRNAs Total RNAs were extracted from arsenic-induced transformed cells and control cells, and miRNA array was performed for analysis. **(A)** Hierarchical clustering and heat map of the differential represented miRs in arsenite-treated cells. Gene tree representation (left) and heat maps (right) of miRNAs differentially regulated during arsenite treatment (T1, T2, T3 = control samples and AS1, AS2, AS3 = arsenite-treated samples) with a p value≤0.05. Each colored bar represents 1 probe set. Bar colors define the degree of expression (red = over-represented miRs; green = down-represented miRs). **(B)** List of differentially expressed miRNA in AS-TM cells. **(C)** Total RNAs were extracted from arsenic-induced transformed and control cells, and q-RT-PCR was performed to test the target gene expression as indicated. Relative expression levels of the six miRs in control (HaCaT) and arsenite-transformed (AS-TM) cells. Each bar represents the mean ± SEM of three independent replicates. *p value≤ 0.05.

To identify the differential expression of miRNAs induced by arsenite, we analyzed RNA from arsenite-induced transformed cells using microarray. The results showed dramatically changes in twenty-six miRNAs in arsenic-treated HaCaT cells compared with control cells (Figure 2A and 2B). To validate the microarray results, we selected miRNAs with fold differences> 2 or <0.5 with priority verified p <0.01. Six miRNAs were selected for further analysis via q-RT-PCR. The results showed that mir-6739-5p, mir-4521, mir-181b-5p, mir-100-5p and hmir-3919 were up-regulated, while hsa-mir-513a-5p was down-regulated in arsenic-treated HaCaT cells (Figure 2C).

### Comparative proteome analysis in arsenic-induced transformed cells

**Figure 3:**
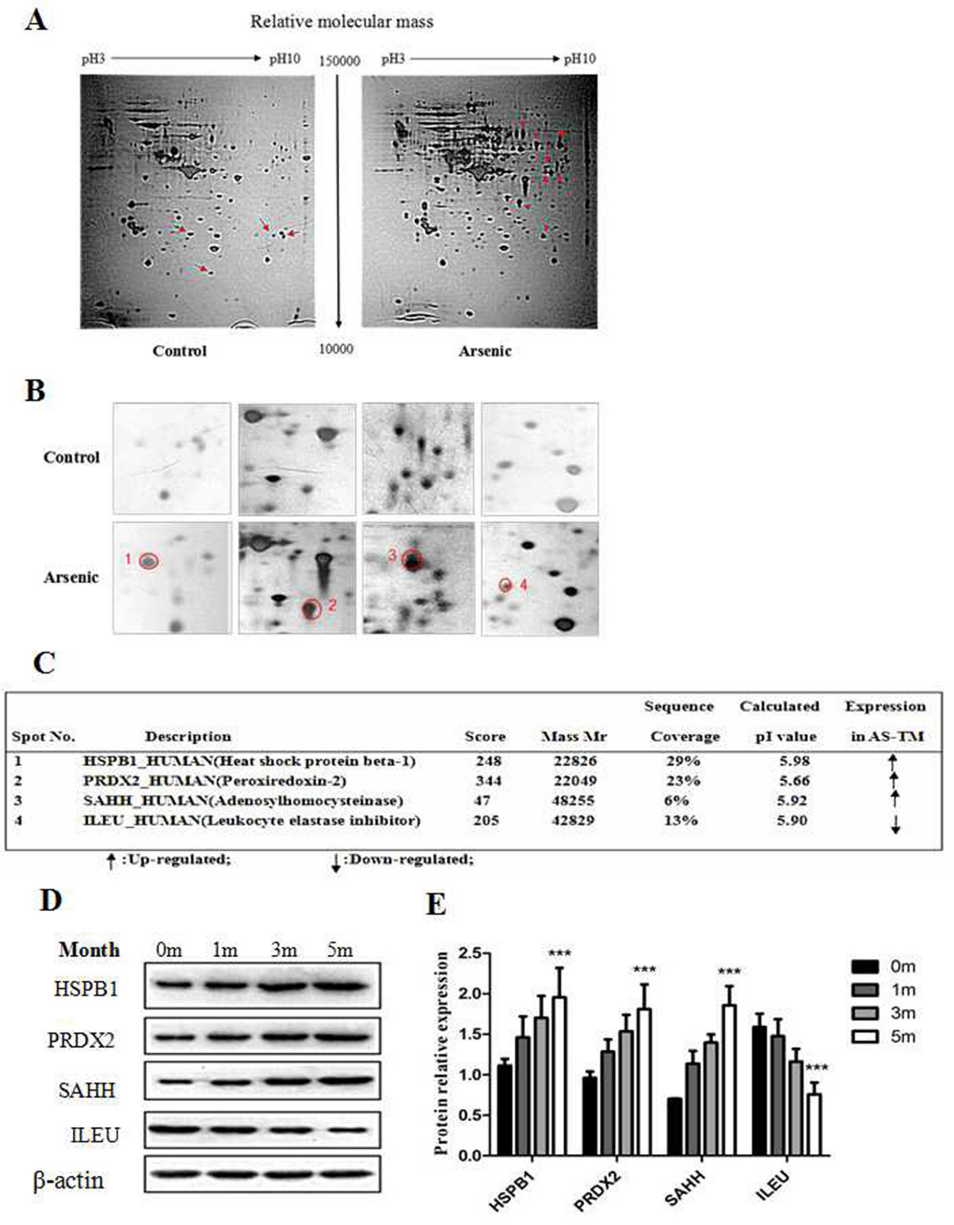
Comparative proteome analysis after arsenic treatment in HaCaT cells HaCaT cells were continuously maintained in DMEM medium containing 100 nM of sodium arsenite for 28 weeks, and subsequently 2D-PAGE plus MS/MS were performed to analyze the protein expression profile. **(A)** Representative 2D gel profiles of total proteins (silver staining). The corresponding arrows show the differentially expressed protein spots, where the left four arrows are the up-regulated protein spots in the control group, and the right eight arrows are the up-regulated protein spots in the arsenic treated group. The details of 2-DE procedure are described in the *Materials and Methods* section. **(B)** Magnification shows the differentially expressed proteins. **(C)** The differential expression of protein spots was identified using mass spectrometry, and the MS/MS procedure is described as in the *Materials and Methods* section. **(D)** The protein levels of HSPB1, PRDX2, SAHH and ILEU were detected using Western blotting. Relative expression levels of HSPB1, PRDX2, SAHH and ILEU in control (0 month) and at different time points (1, 3 and 5 months) of arsenic treatment in HaCaT cells. **(E)** Histograms showing the relative fold-change of HSPB1, PRDX2, SAHH and ILEU protein (mean ± SEM of triplicates) using β-actin as a control for protein loading. ***p < 0.001 vs. Control.

Next, two-dimensional gel electrophoresis (2D-PAGE) coupled to mass spectrometry (MS) was performed to identify the differentially expressed proteins in HaCaT human keratinocytes following exposure to arsenic. As shown in Figure 3A and 3B, more than 200 spots were detected in the 2D maps. Of which, 12 significant differentially expressed spots (8 up-regulated and 4 down-regulated) were identified using LC-ESI-LIT-MS/MS, and alterations in some spots in an arsenic treatment time-dependent manner were confirmed by Western blotting (Figure 3D-3E). These molecules, including heat shock protein beta-1 (HSPB1), peroxiredoxin-2 (PRDX2), adenosylhomocysteinase (SAHH), and leukocyte elastase inhibitor (ILEU), were associated with oxidative stress-induced tumorigenesis.

### UPLC/Q-TOF MS multivariate statistical analysis

**Figure 4:**
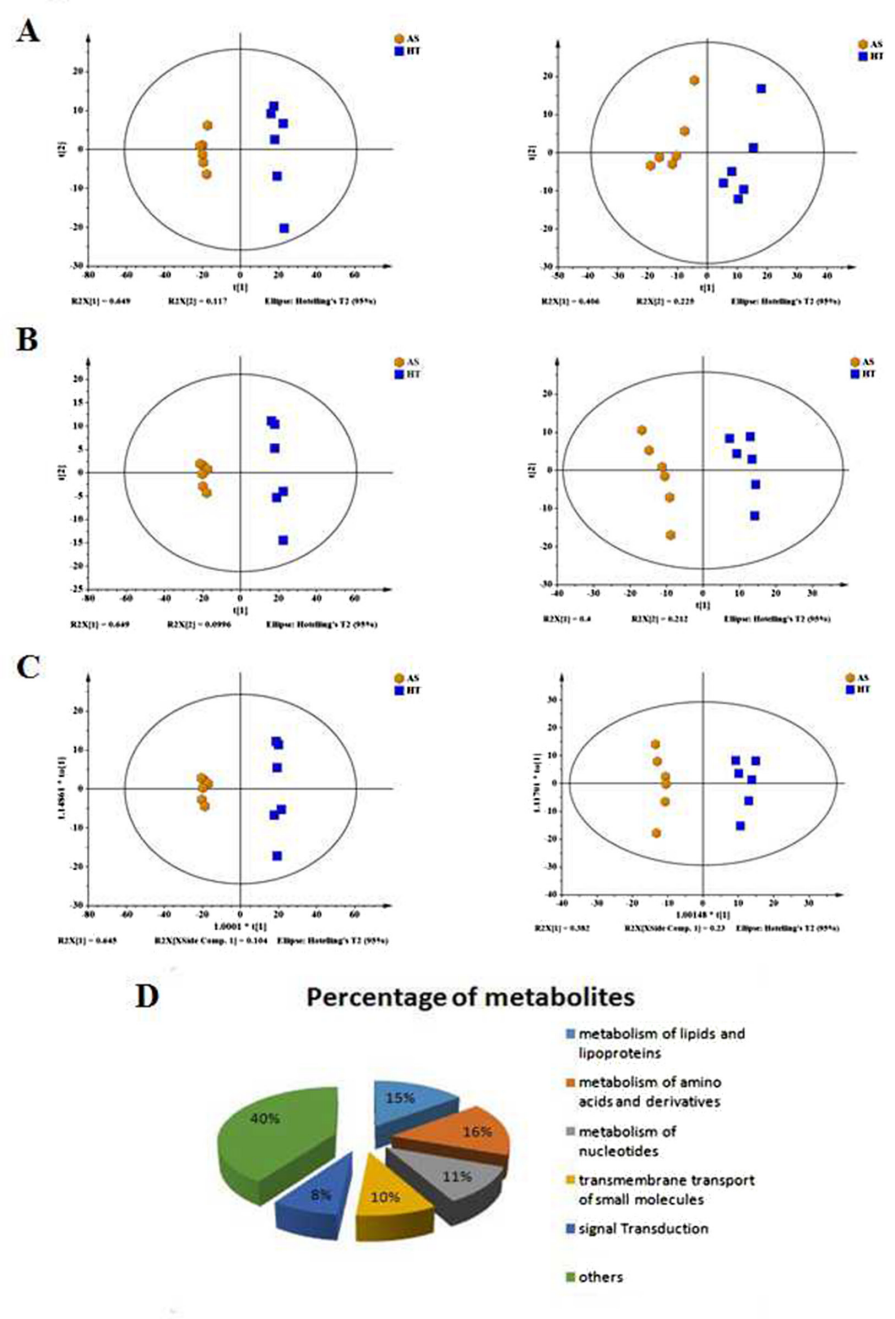
Scoring plots with PCA, PLS-DA and OPLS models of metabolites in control (HT, blue) and arsenic treatment (AS, yellow) **(A)** PCA for the positive model (Left panel); PCA for the negative model (Right panel). **(B)** PLS-DA for the positive model (Left panel); PLS-DA for the negative model (Right panel). **(C)** OPLS for the positive model (Left panel); OPLS for the negative model (Right panel). **(D)** The overview shows the differential metabolites in arsenic-induced transformed cells compared with control cells.

To examine the products and most downstream representation of cellular processes, cells transformed by arsenic were analyzed using UPLC/Q-TOF MS. PCA was initially performed on the datasets to visualize general clustering trends among the observations. Using a PCA score plot (Figure 4A), two principal components (PC), R^2^X=0.766 and Q^2^=0.588 were obtained in positive ESI mode, and R^2^X=0.632 and Q^2^=0.361 were obtained in negative ESI mode. In general, an R^2^X value greater than 0.4 indicates that the model was reliable, thus the model established in this experiment can be applied to visually observe the difference between the two groups of metabolic profiles. The PCA showed no abnormal samples (all samples were within the confidence interval). Therefore, a PLS-DA model was used to further identify the differences among different groups. The parameter R^2^Y represented the interpretation rate of the model, and Q^2^ represented the prediction rate of the model. Generally, the reliable model requires a parameter greater than 0.4. The results showed that two principal components obtained in positive and negative modes, respectively (R^2^X= 0.749, R^2^Y=0.996, Q^2^=0.986 in positive ESI mode, and R^2^X= 0.612, R^2^Y=0.98, Q^2^=0.947 in negative ESI mode) (Figure 4B). Furthermore, the OPLS model was obtained after filtering the orthogonal signal with the model uncorrelated signal. The results showed that one principal component and one orthogonal component were obtained in positive and negative modes, respectively (R^2^X=0.749, R^2^Y=0.996, Q^2^=0.986 in positive ESI mode, and R^2^X=0.612, R^2^Y=0.98, Q^2^=0.995 in negative ESI mode) (Figure 4C).

### Metabolic biomarker screening and identification

The extracted variables that contributed the most to group distinctions were selected as the biomarkers for arsenic exposure. Strict criteria were adopted for screening: (1) variables with a variable importance in the projection (VIP) value>1 were included in the superset of biomarkers; (2) the difference in the candidate level was statistically significant (p <0.05, t-test) between the control and treatment groups; and (3) the online database (http://metlin.scripps.edu/) (more accurate molecular mass) was searched to characterize differential metabolites. Following these criteria, 68 altered metabolites were identified and considered as potential biomarkers (Figure 4D) (Tables 1 and 2), among, which 27 metabolites were increased, while 41 were decreased after arsenic treatment.

**Table 1:** Differential metabolites in HaCaT exposed to arsenic (ESI+)

VIP	[H+]mz	rt (min)	Name	ttest	fold (AS/HT)
1.114	204.1230	0.75	Acetylcarnitine	0.000	-2.400
1.061	146.1158	3.65	Acetylcholine	0.000	-1.432
1.071	268.1043	1.10	Adenosine	0.000	-2.029
1.173	305.2477	13.37	Arachidonic Acid	0.000	-4.060
1.227	304.2616	13.41	Arachidonoyl amine	0.000	4.840
1.238	335.2948	12.96	C22:3	0.000	7.624
1.217	162.1123	0.71	Carnitine	0.000	-1.950
1.090	255.2316	13.25	cis-9-palmitoleic acid	0.000	-2.158
1.058	132.0767	0.73	Creatine	0.001	-0.984
1.219	200.2008	10.59	dodecanamide	0.000	3.166
1.216	154.0831	1.24	Dopamine	0.000	-2.942
1.124	307.2609	0.63	Eicosatrienoic acid	0.000	-0.955
1.050	152.0569	1.19	Guanine	0.001	-2.732
1.040	284.0990	1.19	Guanosine	0.001	-2.751
1.001	137.0457	1.05	Hypoxanthine	0.002	-1.107
1.151	255.0863	5.65	L-Arginine phosphate	0.000	-0.879
1.236	132.1012	1.23	Leucine/Isoleucine	0.000	-2.357
1.230	280.2634	12.66	Linoleamide	0.000	4.231
1.156	279.2295	0.64	Linolenic Acid	0.000	-1.151
1.201	150.0583	1.05	Methionine	0.000	-3.080
1.056	664.1165	1.05	NAD	0.001	-1.513
1.219	282.2808	13.41	Oleamide	0.000	2.667
1.230	256.2639	13.16	Palmitic amide	0.000	3.979
1.210	254.2480	12.28	Palmitoleamide	0.000	4.255
1.159	220.1178	2.66	Pantothenic Acid	0.000	-3.051
1.086	496.3400	10.99	PC(16:0)	0.000	-1.805
1.083	518.3217	10.99	PC(18:3)	0.000	-1.767
1.033	526.2929	10.41	PE(22:6)	0.001	-2.555
1.144	194.0811	5.12	Phenylacetylglycine	0.000	-4.265
1.226	166.0857	2.07	Phenylalanine	0.000	-2.569
1.156	318.2999	8.40	Phytosphingosine	0.000	-2.090
1.095	116.0704	0.73	Proline	0.000	-1.067
1.092	146.1650	0.60	Spermidine	0.000	-2.636
1.102	302.3055	9.26	Sphinganine	0.000	-1.804
1.195	300.2899	9.58	Sphingosine	0.000	-2.448
1.009	284.2942	14.22	Stearamide	0.001	3.746
1.197	205.0972	3.84	Tryptophan	0.000	-2.670
1.201	182.0812	1.10	Tyrosine	0.000	-2.737
1.236	118.0864	1.05	Valine	0.000	-2.501
1.174	153.0420	1.06	Xanthine	0.000	1.819

Fold (AS/HT) represents the relative level of the metabolite abundance in the arsenic treated group. A positive sign indicates a rise in the arsenic treated group (AS) relative to the control group (HT), with a negative sign indicating a decrease.

**Table 2:** Differential metabolites in HaCaT exposed to arsenic (ESI-)

VIP	[H-]mz	rt (min)	Name	ttest	fold (AS/HT)
1.001	131.0705	4.65	2-Hydroxycaproic acid	0.035	0.688
1.097	322.0446	0.71	5'-CMP	0.017	2.172
1.455	338.9895	0.86	D-Fructose 1,6-bisphosphate	0.000	1.691
1.409	303.2339	13.39	Arachidonic Acid	0.000	1.375
1.051	242.0802	0.71	Cytidine	0.008	1.102
1.357	346.0570	0.75	deoxyguanosine 5'-monophosphate (dGMP)	0.001	1.585
1.439	498.9073	0.61	D-myo-Inositol-tetraphosphate	0.000	-1.243
1.306	115.0036	0.76	Fumaric acid	0.002	-1.051
1.044	306.0775	0.76	Glutathione	0.026	1.163
1.176	214.0494	0.70	Glycerylphosphorylethanolamine	0.009	1.749
1.178	362.0516	0.76	Guanidylic acid (guanosine monophosphate)	0.001	1.451
1.284	282.0854	1.19	Guanosine	0.003	-1.951
1.306	154.0624	0.68	Histidine	0.002	-0.978
1.491	186.1141	5.22	KAPA	0.000	1.672
1.018	526.2943	10.68	LysoPE(22:5)	0.031	-1.211
1.259	133.0104	0.76	Malic acid	0.003	-0.880
1.055	194.0829	9.88	n-acetyldopamine	0.024	0.389
1.339	742.0681	1.06	NADP+	0.001	0.772
1.220	281.2493	14.16	Oleic Acid	0.005	0.946
1.468	218.1041	2.66	Pantothenic Acid	0.000	-2.145
1.175	524.2792	10.42	PE(22:6)	0.009	-1.454
1.479	192.0673	5.12	Phenylacetylglycine	0.000	-3.805
1.040	176.9365	0.73	Pyrophosphate	0.026	1.011
1.271	606.0748	0.86	UDP-N-acetyl-D-galactosamine	0.003	2.880
1.256	402.9958	0.89	Uridine diphosphate	0.004	1.144
1.379	323.0297	1.05	Uridine monophosphate (UMP)	0.000	1.226
1.106	151.0266	1.06	Xanthine	0.000	1.896
1.042	179.0566	0.69	α-D-Glucose	0.026	-0.811

Fold (AS/HT) represents the relative level of the metabolite abundance in the arsenic treated group. A positive sign indicates a rise in the arsenic treated group (AS) relative to the control group (HT), with a negative sign indicating a decrease.

The metabolic pathways involved in the differential metabolites were analyzed using the Reactome Pathway Database (http://www.reactome.org/). The software generated 26 metabolic pathways considered significantly associated with arsenic-induced metabolic changes. These 26 pathways were characterized as redox metabolism, beta-alanine metabolism, citrate cycle (TCA cycle), glutathione metabolism, glycerophospholipid metabolism, nicotinate and nicotinamide metabolism, phenylalanine tyrosine and tryptophan biosynthesis, phenylalanine metabolism, purine metabolism, sphingolipid metabolism, tyrosine metabolism, etc. Briefly, redox metabolism, glutathione metabolism and amino acid biosynthesis and metabolism are the major metabolic pathways disrupted after arsenic exposure.

### Molecular networks and metabolism pathways involved in arsenic treatment

**Figure 5:**
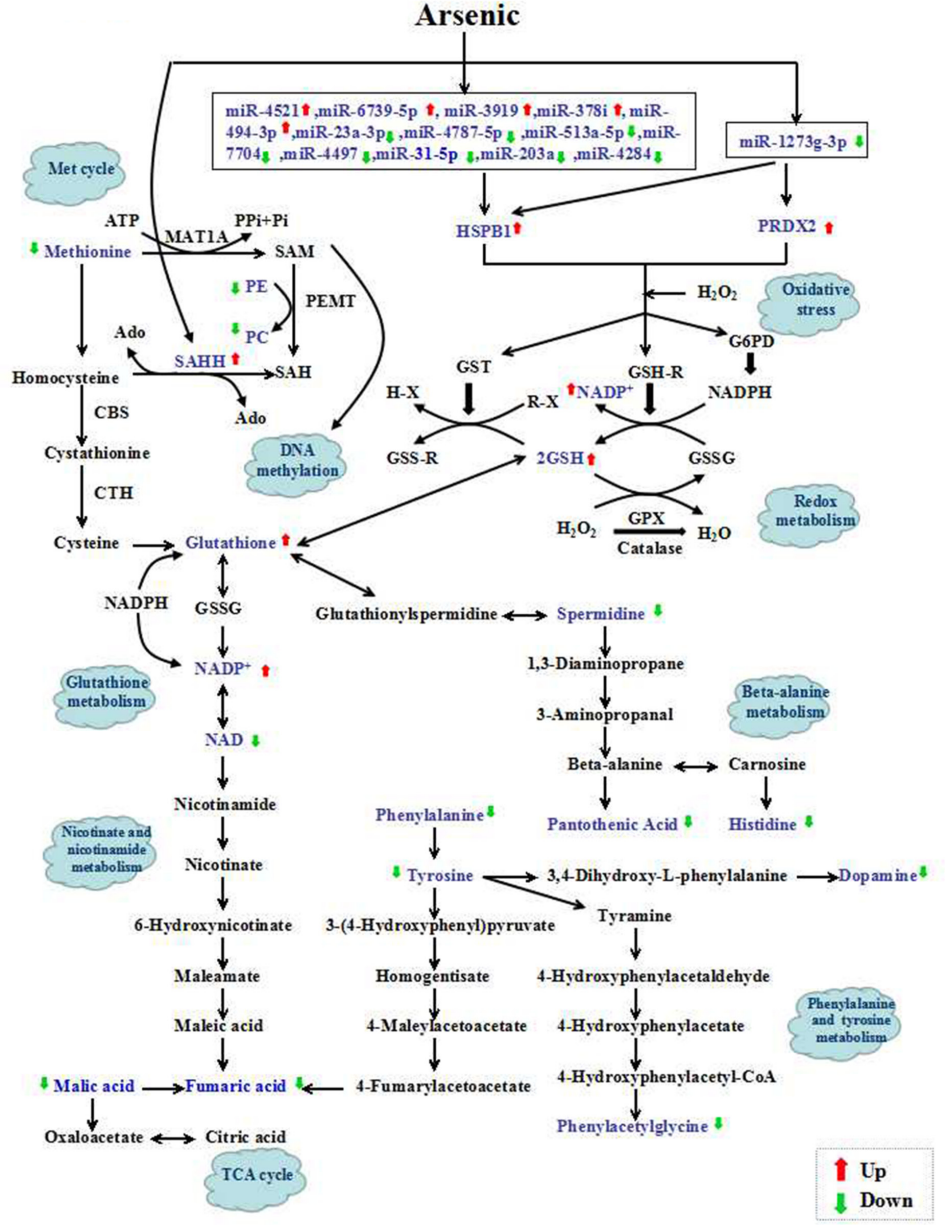
Schematic overview of the integrated the microRNAome, proteomics and metabolomics data to analyze arsenic-induced malignant transformation The metabolites identified in the present study are highlighted in blue. The up- and down-regulation of metabolites is shown with red and green arrows, respectively. GSH=glutathione; G6PD=glucose-6-phosphate dehydrogenase; GST=glutathione transferase; GPX=glutathione peroxidase; GSSG=oxidized GSH; CBS =cystathionine β-synthase; CTH= cystathionine β-lyase; PPi = pyrophosphate; Pi = inorganic P; SAM = S-adenosylmethionine; PE = phosphatidylethanolamine; PC = phosphatidylcholine; SAH = S-adenosylhomocysteine; Ado = adenosyl; PEMT=phosphatidylethanolamine methyltransferase.

To investigate whether the differential miRNAs, proteins and metabolites interacted biologically, the Reactome Pathway Database (http://www.reactome.org/), KEGG Pathway Database (http://www.genome.jp/kegg/pathway.html), miR2Subpath Database (http://202.97.205.78:8080/miR2Subpath/index.jsp), and TargetScan (www.targetscan.org) software were used to build networks to elucidate the signaling pathways impacted by arsenic in HaCaT. As shown in Figure 5, 14 miRNAs (particularly miR-1273g-3p), 3 proteins (HSPB1, PRDX2 and SAHH) and 5 metabolites (glutathione, NADP^+^, methionine, PE and PC) were associated with oxidative stress, glutathione metabolism, met cycle and DNA methylation. Furthermore, 11 metabolites were associated with phenylalanine tyrosine and tryptophan biosynthesis and metabolism, nicotinate and nicotinamide metabolism, citrate cycle (TCA cycle) and beta-alanine metabolism, including fumaric acid, malic acid, citric acid, phenylalanine, tyrosine, spermidine, pantothenic acid, phenylacetylglycine, histidine and dopamine. Moreover, glutathione metabolism is the key pathway of this network, suggesting the critical role of redox in the arsenic-induced malignant transformation of human keratinocytes.

## DISCUSSION

Arsenic (As) is a widely distributed environmental toxicant that causes skin cancer [45]. The types of skin cancer associated with arsenic include intraepidermal carcinomas (Bowen disease) [46], squamous cell carcinomas (SCC), basal cell carcinomas (BCC) [47], Merkel cell carcinoma (MCC) [48] and head and neck cancers [49]. Microarray gene expression has revealed that arsenic impacts the function of diverse tissue types, including the skin [50, 51], bladder and kidney [52], liver [53], prostate and lung [54], peripheral lymphocytes [55], neural tube [56], and urogenital cells [57]. HaCaT cells are non-tumor-derived immortalized normal human keratinocytes cell lines with non-tumor properties. Previous studies have confirmed that the continuous exposure of HaCaT cells to low concentrations of arsenic for approximately 28 weeks induces malignant transformation, manifested as the formation of multinucleated giant cells and highly aggressive squamous cell carcinoma after inoculation into nude mice, indicating that these cells obtain a high degree of malignant phenotypes [58–61]. Pi et al. [62] showed that HaCaT cells exposed to low concentrations (100 nM) of sustained excessive arsenic trioxide show abnormal cell proliferation, morphological changes, apoptosis resistance, increased cloning, and uncontrolled cell growth. Sun et al. [63] confirmed that HaCaT cells exposed to the same concentration of arsenic for 20 weeks showed multinucleated giant cell formation and increased clonogenic formation, indicating that HaCaT cells exposed to arsenic for 20 weeks undergo malignant transformation. Arsenic induces a highly malignant cell phenotype and arsenic poisoning, replicating most common skin squamous cell populations. Arsenic-induced HaCaT cells acquire a malignant phenotype and can be used to simulate arsenic-induced skin malignancy. The molecular mechanism of arsenic-induced skin cancer using this HaCaT cell malignant phenotype as a cellular model has been recognized. Clearly, arsenic affects diverse tissue types. However, currently, there are no reports on the combined microRNAome, proteomics and metabolomics analyses of arsenic-induced malignant transformation of HaCaT cells, thus an understanding of the molecular mechanisms of arsenic-induced cancers in diverse tissue types is needed. Therefore, here we established an arsenic-induced HaCaT cell model based on the results of previous studies for the experimental group, and used normal HaCaT cells as a control group. Notably, the arsenic concentration used in the present study was comparable to human the blood arsenic levels observed in chronic arsenosis patients in Inner Mongolia, China, where arsenic-induced skin lesions and cancers are common [64]. A combined approach of microRNAome, proteomics, and metabolomics profiling was used to determine alterations in the levels of miRNAs, proteins and metabolic substances in HaCaT keratinocytes in response to arsenic exposure.

MiRNAs are a large family of endogenously produced short single-stranded (approximately 22 nucleotides) non-coding RNA molecules transcribed in animals, plants and some viruses, which suppress target gene translation through binding to mRNA [65]. In addition, miRNAs served as versatile gene expression regulators in virtually all cellular pathways in higher eukaryotes, including proliferation, apoptosis, cell differentiation, cell cycle, embryonic development and cellular disease [66, 67]. Additionally, miRNA expression is altered in cancer emergence and metastatic occurrences, therefore playing a pivotal role in health and disease [65]. In the present study, HaCaT cells exposed to 100 nM arsenite for 28 weeks were identified as malignant based on the observation of anchorage-independent growth. Moreover, giant multinuclear cells were observed during the transformation. MiRNA microarray was used to screen differentially expressed miRNAs in two groups of cells, and the results revealed a total of 26 miRNAs (12 up-regulated and 14 down-regulated) differentially expressed in arsenic-treated cells compared to untreated controls. Among of these miRNAs, mir-4521, mir-181b-5p, mir-100-5p and mir-3919 were significantly up-regulated, and mir-513a-5p was down-regulated. Additional findings confirmed that these six miRNAs were indeed up-regulated and down-regulated in arsenic-stimulated keratinocytes according to quantitative PCR assay. These results suggest that arsenic induced the differential expression of miRNAs during the malignant transformation of HaCaT cells, and miRNAs may be involved in the carcinogenesis and development of arsenic-induced skin diseases.

As a next step, we investigated the protein expression profile using high-resolution 2-DE combined MS and confirmed heat shock protein beta-1 (HSPB1), peroxiredoxin-2 (PRDX2), adenosylhomocysteinase (SAHH), and leukocyte elastase inhibitor (ILEU) were dramatically changed in arsenic-induced transformed cells. The majority of the identified proteins were associated with antioxidation and tumorigenesis.

Heat shock protein 27 kDa (HSPB1) is associated with stress responses and is typically expressed during tissue remodeling. The major function of this protein is to stabilize protein structure, and HSPB1 is also involved in decreasing the intracellular reactive oxygen species (ROS) in a glutathione-dependent manner [68]. Thus, HSPs in particular, play a protective role against the deleterious effects of redox imbalance observed when ROS production exceeds scavenging mechanisms. HSPB1 is implicated in maintaining the equilibrium or redox homeostasis by maintaining the level of glutathione, a major redox mediator [69]. Aldrian et al. [70] confirmed that HSPB1 inhibits the proliferation of malignant melanoma cells and reduces the tumorigenicity of malignant melanoma cells in nude mice, suggesting that HSPB1 may affect the tumor cell phenotype. Li GP et al. [71] reported that the expression of HSPB1 was associated with nasopharyngeal carcinoma cell differentiation programs, and HSPB1 was highly expressed in poorly differentiated cancer cells. Moreover, the high expression of NF-κB and MMPs has also been demonstrated in poorly differentiated nasopharyngeal carcinoma cell lines with high expression of HSPB1, suggesting that HSPB1 might enhance the invasion, proliferation and migration of nasopharyngeal carcinoma cells through the activation of the NF-KB signaling pathway and increased expression of MMPs.

Peroxiredoxin-2 (PRDX2) belongs to a superfamily of antioxidant proteins. Johnson et al. [69] showed that the quantitative modeling of H_2_O_2_ metabolism in GPx-deficient mouse erythrocytes required a substantial but not over-whelming contribution of PRDX2. Low et al. [72] confirmed the relevance of PRDX2 in H_2_O_2_ consumption in human erythrocytes. PRDX2 also protects hemoglobin against aggregation [73] and binds many proteins, some of which are bound in a redox-dependent manner [74–77]. A number of recent studies have shown that PRDX2 is involved in tumor proliferation and differentiation [78], associated with signaling pathways within apoptotic cells [79]. Noh et al. [80] showed that PRDX2 overexpression in breast cancer may be associated with the occurrence and development of tumors.

Adenosylhomocysteinase (SAHH) is a conserved enzyme that catalyzes the hydrolysis of S-adenosylhomocysteine (SAH) to adenosine (Ado) and homocysteine (Hcy) in eukaryotes. This conversion is the main route for the breakdown of SAH as a product inhibitor of S-adenosylmethionine (SAM)-dependent methylation reactions. The inhibition of this enzyme increases SAH accumulation, inhibiting the methylation pathway via a feedback inhibition mechanism. In 2008, SAHH was identified as a novel tumor suppressor using genome-wide loss-of-function genetic screening and its down-regulation at mRNA and protein levels was detected in a considerable percentage of different tumor types, including colon and lung cancers [81]. However, as a novel tumor suppressor gene, SAHH overexpression in skin squamous cell carcinoma has not been reported. In the present study, SAHH was associated with DNA methylation and glutathione metabolism.

Leukocyte elastase inhibitor (ILEU), also known as SERPINB1, has been associated with the occurrence and development of various tumors. Chou and others have shown that SERPINB1 may play a role as a tumor suppressor in breast and lung cancers [82].

Oxidative stress occurs when an imbalance exists between the production of reactive oxygen metabolites and the neutralizing availability of antioxidants. Some well-established antioxidants include glutathione, superoxide dismutase (SOD), and vitamins A and E. Heavy metals could cause oxidative stress by disturbing the balance of free radicals [83]. Many metals and metalloids can trigger reactive oxygen species (ROS) production, disrupt signal transduction, alter gene expression, and induce lipid and deoxyribonucleic acid (DNA) damage [84]. Major As-induced ROS include superoxide anion (O_2_^**·**—^), hydroxyl radical (^**·**^OH), hydrogen peroxide (H_2_O_2_), singlet oxygen, and peroxyl radicals. Oxygen-derived radicals are the most important class of radical species generated in living systems as a result of the particular electronic configuration of molecular oxygen, leading to the formation of singlet oxygen through the addition of a single electron. Superoxide anions, resulting from metabolic processes or oxygen “activation” through physical irradiation, are considered “primary” ROS molecules that further interact directly through enzyme-catalyzed or metal-catalyzed processes with other molecules to generate “secondary” ROS products. Reactive oxygen species adversely affect cellular proteins, DNA, and membrane lipids and stimulate an increase in antioxidant defense. Furthermore, As-induced^**·**^OH generation has also been reported in the striatum of rats. Apart from direct evidence of As-induced ROS, indirect evidence has also been reported. Arsenic exposure generates ROS in the cells of fish and mammals [85]. Consistent with previous studies, the data in the present study showed that the miRNAs level of Hsps and protein expression of HSPB1, PRDX2 and SAHH were up-regulated in a time-dependent manner in HaCaT cells after exposure to As_2_ O_3_ compared with the control (p < 0.05), suggesting that excess ROS is generated in HaCaT cells under arsenic exposure, and the antioxidant capacity was restrained compared with the control after exposure to arsenic.

Glutathione is the most abundant nonprotein thiol in defense against oxidative stress. The importance of the antioxidant activity of enzymes, such as glutathione peroxidase, during the peripartal period has been well established [86]. The enzyme glutamate-cysteine ligase (GCL) is rate limiting in the synthesis of glutathione, and is detected as a heterodimeric complex comprising a catalytic (GCLC) subunit and modifier (GCLM) subunit. The last step in the synthesis of glutathione is mediated through GSS, which binds glycine to theγ-glutamylcysteine complex through GCLC [87]. Increased Met bioavailability through Met supplementation can indirectly increase the production of total hepatic glutathione through the transsulfuration pathway [88], and oxidative stress is among the many conditions that could increase GCL activity and GCLC mRNA expression [89]. The results of the present study suggested that HSPB1, PRDX2 and SAHH were associated with glutathione metabolism in the malignant transformation of HaCaT cells induced by arsenic, and the mechanism of these proteins and the metabolism pathway needs further study.

In conclusion, through a combined microRNAome, proteomics and metabolomic analysis, the present study investigated the effects of arsenic exposure on miRNA and protein expression and metabolic pathways in HaCaT cells. A series of differential miRNAs, proteins and metabolites particularly associated with oxidative stress was identified. In addition, we suggested that the dysregulation of miRNAs (particularly miR-1273g-3p), proteins (HSPB1, PRDX2 and SAHH) and metabolites (glutathione, NADP+, methionine, PE and PC) induced arsenic prevents oxidative damage, which was involved in the activation of glutathione metabolism pathways (Figure 5). These findings could be extended to more details of networks induced by arsenic and their inference in carcinogenesis, which may provide the foundation for developing better therapeutic strategies.

## MATERIALS AND METHODS

### Cell culture and arsenate exposure

Human keratinocyte HaCaT cells (maintained in the lab) were cultured in DMEM supplemented with 10% FBS, 100 U of penicillin/ml, and 100 mg of streptomycin/ml. The cultures were maintained at 37°C in a humidified 5% CO_2_ atmosphere. The culture media was replaced with fresh growth media every 2–3 days.

For chronic arsenic exposure, the cells were continuously maintained in medium containing 100 nM of sodium arsenite (NaAsO_2_, Sigma, St. Louis, MO) for 28 weeks [44]. All materials for cell culture were purchased from Thermo Scientific HyClone (Logan, UT, USA).

### Anchorage-independent growth assay

The cells (5×10^3^/well) were suspended in 1 mL of DMED medium supplemented with 10% FBS and 0.33% agar, seeded onto 3 mL of solidified DMED medium supplemented with 10% FBS and 0.5% agar in 6-well plates, and subsequently cultured for 3 weeks. The colonies were scored using a microscope and ImageJ computer software.

### Microarray analysis

Three control samples and three experimental samples were sent to the LC-Bio Company (Beijing, China) for miRNA microarray hybridization and analysis. The miRCURY™ RNA Isolation Kit and the miRCURY™ LNA Array power labeling kit (Exiqon) were used to extract total RNA and label the samples. The samples were subsequently scanned using the Agilent G2565BA Microarray Scanner System (Santa Clara, CA). ImaGene 8.0 software was used for the image analysis (BioDiscovery, Inc., Hawthorne, CA). Normalization was performed using the global Lowess regression algorithm, the original data was shown in Supplementary Table 1.

### MiRNAs isolation and cDNA preparation

To validate the microarray data, a miRNA purification kit (Biosynthesis, Lewisville TX) was used to extract miRNAs from the remaining biological replicates of the untreated control (N=3) and arsenite-treated (N=3) samples. Subsequently, cDNA was synthesized for each sample using the miRNA First-Strand synthesis kit following manufacturer’s instructions (Clontech, Mountain View, CA).

### Quantitative real-time PCR analysis

MiRNA primers, used to amplify the entire sequence, were designed for the six selected miRNAs (Integrated DNA technologies Inc., Coralville, IA). The miRNA expression was quantified using a SYBR qRT-PCR kit (Clontech) and the cDNA from each of the three biological replicates of arsenite-treated (N=3) and control samples (N=3). U6 was used as a reference gene. The forward primers were designed and synthesized at the BIONEER Company (Korea), and the reverse primers were provided in the reagent kit. The following forward primers were used:

**Table:** 

miRNA	Sequence
mir-181b-5p	5’-GCAACATTCATTGCTGTCGGTGGGT-3’
mir-100-5p	5’-GCGCAACCCGTAGATCCGAACTTGT-3’
mMir-6739-5p	5’-GCGCAGTGGGAAAGAGAAAGAACAAGT-3’
mir-4521	5’-GCGCTAAGGAAGTCCTGTGCTCAG-3’
mir-3919	5’-GGGCAGAGAACAAAGGACTCAGT-3’
mir-513a-5p	5’-GCGTTCACAGGGAGGTGTCAT-3’
U6	5’-ACACGCAAATTCGTGAAGCGTTCC-3’

### Functional clustering of miRNAs putative target

To obtain a wide view of the potential miRNA targets, we performed an in silico prediction analysis using three distinct software: Miranda (www.microrna.org), TargetScan (www.targetscan.org) and PicTar (www.pictar.org). The target genes, predicted by above-mentioned programs, were matched using MatchMiner software (www.discover.nci.nih.gov/matchminer/index.jsp).

### 2-DE and image analysis

Two-dimensional electrophoresis (2-DE) and gel-image analysis were performed as reported in the literature [90]. Briefly, total proteins (600 mg) were loaded onto 24 cm IPG strips (immobilized pH 4-7; GE Healthcare, Waukesha, WI, USA) by rehydration loading overnight. Isoelectric focusing was performed at 50 V for 4 h, 100 V for 1 h, 500 V for 1 h, 1000 V for 1 h, 2000 V for 1 h, 4000 V for 2 h, 8000 V for 5 h, 8000 V for 9 h, and 50 V for 6 h using an IPGphor II platform (GE Healthcare). Each focused strip was equilibrated in 5 ml of equilibration buffer containing 6 M urea, 30% (v/v) glycerol, 2% (w/v) SDS, 50 mM Tris-HCl (pH 6.8), 0.1 mg/ml of bromophenol blue, and 100 mM DTT as a first step, and 55 mM iodoacetamide as a second step. The two-dimensional separation of proteins was performed on 10% SDS-PAGE at 2 W per gel, 500 V, and 300 mA for 30 min, followed by 16 W per gel, 700 V, and 300 mA. The gels were stained with colloidal CBB (Coomassie brilliant blue) G-250 staining solution [34% (v/v) methanol, 17%(w/v) ammonium sulfate, 3% (v/v) phosphoric acid, and 0.1% (w/v) CBB G-250] and destained twice with 30% (v/v) methanol distilled water. The stained gels were scanned for image analysis using a transmission scanner (PowerLook 1120, UMAX, Dallas, TX, USA) with a 32-bit pixel depth and 300 dpi resolution. Protein spots on 2D gels were detected and processed using ImageMaster 2D Platinum software 6.0 (GE Healthcare, Waukesha, WI, USA). The percentage volume of each spot was determined from three biological replicates and the original data was shown in Supplementary Table 2.

### Mass spectrometry analysis and protein identification

The gel spots were excised, S-alkylated, digested with trypsin, and subjected to a desalting/concentration step on μZipTip C_18_ pipette tips. The samples were subsequently analyzed by nanoLC-ESI-LIT-MS/MS using an LTQ XL mass spectrometer (Thermo, San Jose, CA, USA) equipped with a Proxeon nanospray source connected to an EasynanoLC (Proxeon, Denmark). Peptide mixtures were separated on an Easy C_18_ column (100 × 0.075 mm, 3 μm) (Thermo, USA) using a acetonitrile gradient containing 0.1% formic acid in aqueous 0.1% formic acid; acetonitrile ramped from 5 to 35% over 24 min and from 35 to 95% over 2 min, at a flow rate of 300 nl/min. The spectra were acquired in the range of 400-2000 *m*/*z*. Acquisition was controlled by a data-dependent product ion scanning procedure over the three most abundant ions, enabling dynamic exclusion (repeat count two and exclusion duration 1 min). Mass isolation window and collision energy were set to 3 *m*/*z* and 35%, respectively.

MASCOT 2.3.02 software (Matrix Science, UK) was used to identify spots from a non-redundant human protein database (UniProt nr06/2013, 12 5229 sequences). NanoLC-ESI-LIT- MS/MS data were searched using a mass tolerance value of 2 Da for precursor ion and 0.8 Da for MS/MS fragments, with trypsin as the proteolytic enzyme, a missed cleavage maximum value of 2 and Cys carbamidomethylation, pyroglutamate formation at N-terminal Gln, and Met oxidation as fixed and variable modifications, respectively. Protein candidates with more than two unique assigned peptides with an individual MASCOT score > 25, corresponding to p ≤ 0.05 for a significant identification, were further evaluated through comparison with their calculated Mr and pI values, using the experimental values obtained from 2-DE.

### Immunoblotting

Immunoblotting was performed as previously described [91]. The following antibodies were used: HSPB1 antibody (Santa Cruz Biotechnology, Santa Cruz, CA, USA), diluted at 1:500; PRDX2 antibody (Santa Cruz Biotechnology), diluted at 1:500; SAHH antibody (Abcam, Cambridge, UK) diluted at 1:1000; and ILEU antibody (Abcam), diluted at 1:1000. The blots were normalized to β-actin (Cell Signaling Technology, Inc., Beverly, MA). The bands were quantified using ImageJ software (v4.18).

### Metabolite analysis by UPLC/Q-TOF MS

The instrumental analysis platform was UPLC-Q/TOF-MS (Agilent, 1290 Infinity LC, 6530 UHD and Accurate-Mass Q-TOF/MS). The injection volume was 4μL and the auto-sampler temperature was 4°C. The processed feature tables were subsequently Pareto-scaled and submitted to SIMCA-P V11.5 software (Umetrics, Uppsala, Sweden) for multivariate statistical analysis. Principal component analysis (PCA) was first performed to discover intrinsic treatment-related clusters within the datasets. Subsequently, partial least-squares discriminant analysis (PLS-DA) was used to improve separation among the groups and screen biomarkers, and differentiated metabolites were extracted according to the VIP value of orthogonal partial least squares (OPLS). The levels of metabolites in the arsenic-treated group (AS) and the control group (HT) were compared using the t-test (p<0.05) of the two samples. Fold-(AS/HT) represents the relative level of the substance in the arsenic-treated group. A positive sign indicates an increase in the AS group relative to the HT group, with a negative sign indicating a decrease. Variable importance in projection (VIP) represents the ability of the extracted variables to discriminate between different groups, and the variables with VIP values greater than 1.0 were included in the preset of biomarkers. The qualitative method of differential metabolites was performed after searching the online database (http://metlin.scripps.edu/) and the original data was shown in Supplementary Table 3.

### Statistical analysis

The data are expressed as the mean ± SEM. Student’s t-test or one-way ANOVA was used to determine the significant differences. p<0.05 was considered statistically significant.

## SUPPLEMENTARY MATERIALS TABLES








