# Supplementary material for: Integration of microRNAome, proteomics and metabolomics to analyze arsenic-induced malignant cell transformation
Source: Oncotarget. 2017 Jun 27;8(53):90879–96. doi: 10.18632/oncotarget.18741 (PMC5710891; doi:10.18632/oncotarget.18741)
Supplement: Supplementary file 1 [file oncotarget-08-90879-s001.pdf]

Integration of microRNAome, proteomics and metabolomics to analyze arsenic-induced malignant cell transformation

SUPPLEMENTARY MATERIALS

Supplementary Table 1:

See Supplementary File 1

Supplementary Table 2:

See Supplementary File 2

Supplementary Table 3:

See Supplementary File 3
